# Does Paying the Same Sustain Telehealth? A Systematic Review of Payment Parity Laws

**DOI:** 10.3390/healthcare14020222

**Published:** 2026-01-16

**Authors:** Alina Doina Tanase, Malina Popa, Bogdan Hoinoiu, Raluca-Mioara Cosoroaba, Emanuela-Lidia Petrescu

**Affiliations:** 1 Department of Professional Legislation in Dental Medicine, Faculty of Dental Medicine, “Victor Babes” University of Medicine and Pharmacy, Eftimie Murgu Square No. 2, 300041 Timisoara, Romania; tanase.alina@umft.ro (A.D.T.); cosoroaba.raluca@umft.ro (R.-M.C.); 2 Research Centre in Dental Medicine Using Conventional and Alternative Technologies, Faculty of Dental Medicine, “Victor Babes” University of Medicine and Pharmacy, Eftimie Murgu Square 2, 300041 Timisoara, Romania; petrescu.emanuela@umft.ro; 3 Department of Pediatric Dentistry, Faculty of Dental Medicine, “Victor Babes” University of Medicine and Pharmacy, Eftimie Murgu Square 2, 300041 Timisoara, Romania; popa.malina@umft.ro; 4 University Clinic of Oral Rehabilitation and Dental Emergencies, Faculty of Dentistry, “Victor Babes” University of Medicine and Pharmacy Timisoara, Eftimie Murgu Square No. 2, 300041 Timisoara, Romania; 5 Interdisciplinary Research Center for Dental Medical Research, Lasers and Innovative Technologies, Revolutiei 1989 Avenue No. 9, 300070 Timisoara, Romania; 6 Department of Prostheses Technology and Dental Materials, Faculty of Dental Medicine, “Victor Babes” University of Medicine and Pharmacy, Eftimie Murgu Square 2, 300041 Timisoara, Romania

**Keywords:** telemedicine, health policy, reimbursement mechanisms, access to health services

## Abstract

**Background and Objectives**: Payment parity laws require commercial health plans to pay for telehealth on the same basis as in-person care. We systematically reviewed open-access empirical studies to identify and synthesize empirical U.S. studies that explicitly evaluated state telehealth payment parity (distinct from coverage-only parity) and to summarize reported effects on telehealth utilization, modality mix, quality/adherence, equity/access, and expenditures. **Methods:** Following PRISMA 2020, we searched PubMed/MEDLINE, Scopus, and Web of Science for U.S. studies that explicitly modeled state payment parity or stratified results by payment parity vs. coverage-only vs. no parity. We included original quantitative or qualitative studies with a time or geographic comparator and free full-text availability. The primary outcome was telehealth utilization (share or odds of telehealth use); secondary outcomes were modality mix, quality and adherence, equity and access, and spending. Because designs were heterogeneous (interrupted time series [ITS], difference-in-differences [DiD], regression, qualitative), we used structured narrative synthesis. **Results:** Nine studies met inclusion criteria. In community health centers (CHCs), payment parity was associated with higher telehealth use (42% of visits in parity states vs. 29% without; Δ = +13.0 percentage points; adjusted odds ratio 1.74, 95% CI 1.49–2.03). Among patients with newly diagnosed cancer, adjusted telehealth rates were 23.3% in coverage + payment parity states vs. 19.1% in states without parity, while cross-state practice limits reduced telehealth use (14.9% vs. 17.8%). At the health-system level, parity mandates were linked to a +2.5-percentage-point telemedicine share in 2023, with mental-health (29%) and substance use disorder (SUD) care (21%) showing the highest telemedicine shares. A Medicaid coverage policy bundle increased live-video use by 6.0 points and the proportion “always able to access needed care” by 11.1 points. For hypertension, payment parity improved medication adherence, whereas early emergency department and hospital adoption studies found null associations. Direct spending evidence from open-access sources remained sparse. **Conclusions:** Across ambulatory settings—especially behavioral health and chronic disease management—state payment parity laws are consistently associated with modest but meaningful increases in telehealth use and some improvements in adherence and perceived access. Effects vary by specialty and are attenuated where cross-state practice limits persist, and the impact of payment parity on overall spending remains understudied.

## 1. Introduction

U.S. telehealth policy uses several levers, including licensure and cross-state practice rules, coverage mandates, modality allowances, network adequacy standards, and reimbursement. Among these, payment parity laws are unusually direct: they require commercial plans to pay the same rate (or use the same fee schedule) for telehealth as for in-person services. Coverage parity laws, in contrast, require health plans to cover telehealth when an in-person service is covered, but they do not specify how much is paid. During the COVID-19 public health emergency (PHE), temporary federal and state flexibilities allowed rapid expansion of remote care across specialties. After the PHE, states diverged in how they made these policies permanent, and Medicare temporarily maintained near-parity payments for many services, further normalizing virtual care in routine practice [1,2,3].

Recent policy compendia show wide variation in payment parity statutes, including which services are covered, whether audio-only visits qualify, and whether requirements apply only to fully insured commercial plans or also to self-funded plans [4,5]. As a result, by late 2024, roughly one-fifth to one-half of states had some form of commercial payment parity, often alongside broader coverage parity requirements [4,5]. These differences in timing and policy design between states create natural experiments that can be used to study whether paying the same rate for telehealth, compared with coverage parity alone, sustains telehealth use, alters the mix of audio-only and video visits, and affects downstream quality, equity, and spending [2,3,4,5,6,7]. Payment parity could shift provider and system behavior via several channels. First, by increasing the expected revenue per virtual encounter, parity can justify continuing investments in virtual infrastructure (platforms, staffing, and training) and support hybrid scheduling after the PHE. Second, parity can influence the modality mix: statutes that include (or exclude) audio-only reimbursement may differentially affect access for populations with limited broadband, language needs, or device barriers—especially in mental health, where tele-psychotherapy is clinically suitable and where payer rules have strongly shaped uptake [8,9,10,11]. Third, effects likely vary by specialty: counseling- and monitoring-heavy services (behavioral health and chronic disease management) may translate payment signals into utilization and adherence gains more readily than exam-dependent procedural fields. Fourth, parity interacts with rules outside reimbursement—especially licensure and interstate compacts—such that payment incentives alone may not overcome cross-state frictions [4,5,11]. Finally, parity may alter quality and equity indirectly by enabling easier follow-up, improving “visit completion,” and reducing patient time and travel costs—mechanisms supported by emerging utilization and patient-experience evidence [8,9,10,11].

General telehealth reviews consistently describe the pandemic-era spike and note that enhanced reimbursement and relaxed rules accelerated adoption, yet most either pool coverage and payment provisions or treat payment parity tangentially. Policy trackers and 50-state surveys catalog statutes but do not quantify outcomes; conversely, payer- or health-system-specific studies often evaluate utilization or process metrics without isolating the payment component of reform [4,5,10,11]. Recent scoping work on telehealth payment more broadly highlights heterogeneous designs, fragmented outcome reporting (utilization vs. quality vs. spending), and a dearth of parity-specific causal studies—especially beyond mental health [10,11].

States continue to revise parity statutes—reconsidering audio-only inclusion, mental-health-only carve-ins, and sunset clauses—while payers weigh concerns about overuse and price setting against access, workforce, and equity goals. Safety-net organizations caution that low virtual reimbursement threatens staffing and access if telehealth is undervalued, whereas employers and plans note that undifferentiated parity could blunt efficiency gains if virtual care is priced identically regardless of setting or overhead [4,5,12]. Evidence from Medicare and state analyses suggests that telehealth use remains concentrated in behavioral health and chronic care, that visit completion and preventive measures can improve with virtual options, and that per-member spending impacts depend on utilization volume, modality mix, and parity assumptions used in budgeting models [2,8,12]. For chronic disease management and cancer survivorship, parity may support more frequent touchpoints and adherence—outcomes with downstream clinical significance—provided parity is paired with guardrails (quality measurement and fraud prevention) and digital inclusion strategies [2,8,12].

From a policy perspective, these outcome domains matter for different reasons. Utilization and modality mixes indicate whether payment parity helps maintain a viable volume of telehealth visits and whether patients can use video or audio-only modalities that match their digital access. Quality and adherence outcomes signal whether telehealth encounters paid at parity support guideline-concordant care, such as chronic disease monitoring. Equity and access measures show who benefits from payment parity, including safety-net settings and patients facing digital or geographic barriers. Finally, expenditure outcomes are essential for understanding whether payment parity leads mainly to substitution of telehealth for in-person care or to additive use that increases total costs.

Accordingly, the objectives were to (i) identify and characterize open-access empirical studies that evaluate state payment parity laws for telehealth; (ii) summarize reported effects on utilization (telehealth share and modality shifts), quality (process measures and adherence), equity (subgroup patterns by payer, race/ethnicity, and rurality), and expenditures where available; and (iii) describe contextual modifiers (coverage parity tiers, licensure and interstate practice limits) and evidence gaps to inform legislative refinement and evaluation design. We emphasize quasi-experimental and comparative designs where possible, and we situate findings within contemporary state policy landscapes from nonpartisan trackers and telehealth resource centers to ensure accurate statutory context and generalizability [4,5,13,14,15].

## 2. Materials and Methods

### 2.1. Protocol and Registration

This study is a PRISMA 2020-reported systematic review with a structured narrative synthesis [16]. The protocol prespecified the question, eligibility criteria, outcomes, and extraction plan and was archived locally prior to screening. The review protocol was registered to Open Science Framework (OSF). The primary outcome was telehealth utilization (share or odds of telehealth use, including estimates from interrupted time series [ITS] and difference-in-differences [DiD] models). Secondary outcomes were modality mix (proportion of video vs. audio-only visits), quality and adherence, equity (differences by payer, race/ethnicity, or rurality), patient-reported access, and spending. Secondary outcomes included workforce perceptions and modality mix (audio-only/video). We defined payment parity as a state law or policy requiring comparable reimbursement for telehealth and in-person services for commercial plans (or explicitly stating equivalent payment), distinguishing it from coverage parity, which mandates coverage but not rate equivalence. We anticipated heterogeneity in policy scope (audio-only inclusion, service carve-outs), implementation dates, and enforcement. We planned a narrative synthesis with structured tables because the outcomes and designs were not sufficiently homogeneous for meta-analysis.

We framed the review question using a PICO structure consistent with systematic review reporting principles [16]. The population comprised U.S. patients, clinicians, and health systems evaluated in ambulatory and selected specialty settings (community health centers, oncology, and behavioral health). The intervention was exposure to state telehealth payment parity laws requiring commercial insurers to reimburse telehealth on the same basis as in-person care, with attention to whether audio-only services were included. Comparators were states with coverage parity only or no parity, and (when reported) relevant policy modifiers such as cross-state practice limits. Outcomes were telehealth utilization (primary; e.g., telehealth share or odds of use) and secondary domains including modality mix (video vs. audio-only), quality/adherence, equity/access measures, and expenditures.

### 2.2. Eligibility Criteria

Inclusion criteria: (1) U.S. empirical quantitative or qualitative study linking a state payment parity law (alone or as a tier vs. coverage parity/no parity) to at least one outcome domain. (2) Original data (claims/EHR/surveys/qualitative) with an identifiable comparator (time, geography, or policy tier) and methods beyond anecdotes. (3) Free full text available (PubMed Central, Scopus, Embase, OA publisher, or official OA PDF). We limited inclusion to studies with freely accessible full texts to ensure replicability by clinicians and policymakers without subscription access. Operationally, we applied free-full-text/open-access filters during database searching where available and re-verified full-text accessibility at the full-text eligibility stage. For records that appeared eligible based on their title/abstract but lacked a free full text, we attempted to obtain manuscripts by (i) searching for author-posted versions (e.g., institutional repositories or preprints) and (ii) emailing the corresponding author (two attempts, separated by ≥7 days). If the full text remained unavailable, the record was excluded at full-text review. (4) English. (5) Publication date in any year up to 26 September 2025. We included multi-policy studies if they reported results stratified by parity tier (payment + coverage vs. coverage-only vs. none) or explicitly modeled payment parity. Exclusion criteria: conference abstracts without full text; editorials/commentaries; narrative overviews without empirical results; studies of Medicare/CMS temporary parity that lacked state-law analysis; non-OA items; studies focused solely on licensure or cross-state practice without reimbursement analysis (unless included as a modifier within a payment-parity model). When multiple OA versions existed (publisher/PMC), we used the most complete.

### 2.3. Information Sources and Search Strategies

On PubMed/MEDLINE, we combined controlled vocabulary and keywords for policy and parity: (“telemedicine” OR “telehealth”) AND (parity OR “payment parity” OR “reimbursement parity”) AND (law OR legislation OR statute OR policy) AND (state OR states) AND (United States). We also ran targeted strings such as (“payment parity” AND telehealth) and (“coverage parity” AND telehealth); we applied the free full-text filter and screened related articles. On Scopus and Web of Science, we used analogous keyword sets within titles/abstracts/keywords, combined with United States OR U.S. OR state and limited to OA where platform filters allowed; otherwise, we verified OA availability on the publisher website or PMC. We also hand-searched reference lists of included studies and relevant OA overviews to avoid missing eligible items (e.g., oncology-specific and CHC-focused analyses, as well as national DiD studies and qualitative FQHC research). In addition, we drew on publicly available 50-state telehealth policy trackers [4,5,11] to summarize the distribution of state commercial payment- and coverage-parity laws for descriptive mapping.

### 2.4. Selection Process

Two reviewers independently screened titles and abstracts from database searches and then full texts of potentially eligible reports, resolving disagreements by consensus. We used a standardized data-extraction form in Microsoft Excel that we piloted on two studies to ensure consistent interpretation of each field. The form captured study identifiers (authors, year), setting and population, policy exposure and comparator groups, design (DiD, ITS, cross-sectional, qualitative), data source and time period, sample size or units of analysis, outcome definitions, and key numeric results (odds ratios, percentage-point differences, adjusted rates), as well as any reported policy modifiers (such as coverage parity tier or cross-state practice rules). When numeric results were not provided in the open-access text or tables, we recorded them as “not reported (NR)” and relied on the qualitative description of the direction of effect where available.

For each outcome, we extracted the study’s reported effect estimate and uncertainty (odds ratio, regression coefficient, percentage-point change, 95% confidence interval, and/or *p*-value). When studies presented descriptive percentages without statistical testing or measures of uncertainty, we report those values as descriptive only and avoid inferential comparisons.

**Figure 1:**
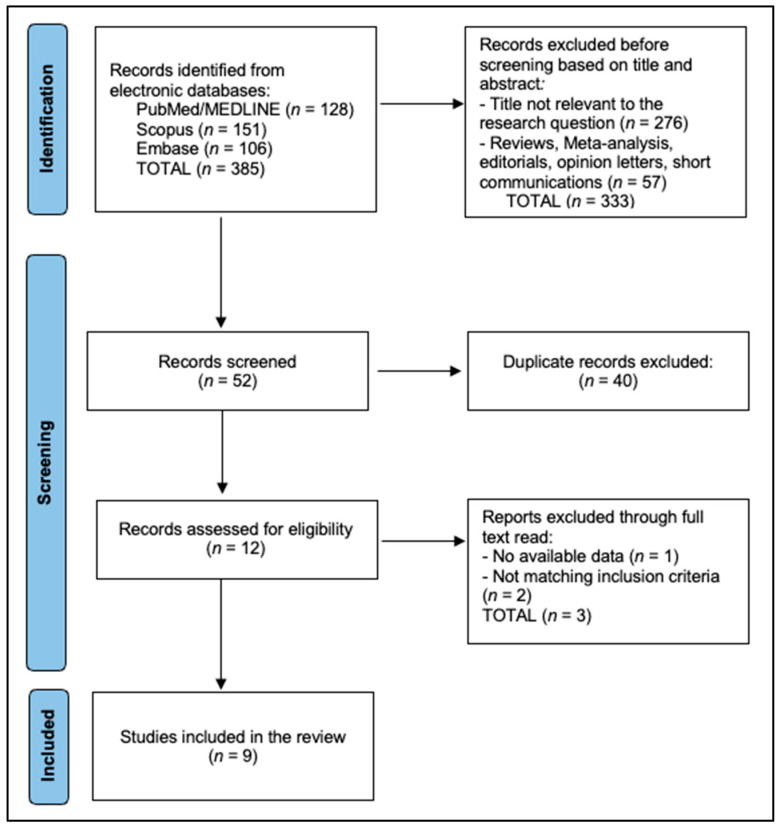
PRISMA flowchart diagram.

The PRISMA flowchart shows that 385 records were initially identified across three databases—PubMed/MEDLINE (*n* = 128), Scopus (*n* = 151), and Embase (*n* = 106). Before formal screening, 333 records were excluded based on title/abstract because they were not relevant to the research question (*n* = 276) or were non-eligible publication types (reviews, meta-analyses, editorials, opinion letters, short communications; *n* = 57). The remaining 52 records proceeded to screening, where 40 duplicates were removed, leaving 12 reports for full-text assessment. At full-text assessment, we confirmed open-access availability and documented any exclusions due to inaccessible full text despite author-contact attempts. Of these, three were excluded after full-text review due to no available data (*n* = 1) or failure to meet inclusion criteria (*n* = 2). Ultimately, 9 studies met all eligibility criteria and were included in the qualitative synthesis, as seen in Figure 1.

### 2.5. Risk of Bias

Given policy heterogeneity and observational designs, we qualitatively appraised risk of bias using domains from ROBINS-I: confounding, selection of participants, misclassification of exposure (policy status), deviations from intended interventions, missing data, outcome measurement, and selective reporting. For each study, two reviewers independently judged each domain as low, moderate, serious, or critical risk, resolving disagreements by discussion; an overall judgment was then assigned following ROBINS-I guidance. Particular attention was paid to unmeasured confounding from concurrent policies, policy misclassification, and secular trends in telehealth adoption.

## 3. Results

### 3.1. Study Characteristics and Policy Context

Table 1 maps nine studies assessing state telehealth payment parity [17,18,19,20,21,22,23,24,25], spanning diverse contexts and methods: emergency departments with cross-sectional/panel analyses of parity presence [17]; community health centers using logistic regression on FAIR Health data (N = 6598) [18]; a large oncology cohort (110,461 total; parity analyses *n* = 53,982) modeled with ITS and multivariable approaches and incorporating cross-state policy limits [19]; a quasi-experimental national claims study examining coverage requirements and related provisions [20]; a hospital-level adoption landscape survey (telehealth capability) [21]; DiD analyses of parity vs. coverage parity vs. none for adults with hypertension [22]; a health-system panel (January 2019–March 2023) linking state-parity mandates to telemedicine share [23]; qualitative interviews with 56 staff across six NYC FQHCs on reimbursement levels and sustainability [24]; and a DiD claims study of privately insured workers (*n* = 29,204; 1,051,344 person-months) evaluating parity’s effect on telehealth and outpatient utilization [25]. Periods cover pre-COVID baselines through late-pandemic stabilization, enabling comparisons across policy eras and plan types. To situate these empirical studies within the broader policy environment, we created a descriptive analysis that classifies states as having commercial payment + coverage parity, coverage-only parity, or no commercial parity at two time points (mid-2020 and mid-2023), using nonpartisan trackers from the Center for Connected Health Policy, the National Conference of State Legislatures, and the American Telemedicine Association [4,5,11].

Across the nine studies, all were judged to have at least a moderate risk of bias in one or more ROBINS-I domains. Confounding from concurrent policies and secular trends was commonly rated as a moderate or serious risk, particularly in pre-pandemic cross-sectional adoption studies. Misclassification of policy exposure and incomplete reporting of outcome measurements were additional concerns in some analyses. No study achieved an overall low-risk judgment, which reinforces the need to interpret the observed parity-associated effects as suggestive rather than definitive.

### 3.2. Telehealth Utilization and Modality Mix

Table 2 presents utilization findings as reported in the included studies, prioritizing effect estimates with uncertainty (95% CI and *p*-values when available). When only descriptive percentages were provided without accompanying statistical testing, we report them descriptively without inferring differences. In CHCs, payment parity correlated with greater telehealth use—42% of sites using telehealth in April 2021 in parity states vs. 29% without (Δ = +13.0 pp), with OR 1.740 (*p* < 0.001) for 2021 site-month use [18]; among newly diagnosed cancer patients, adjusted telehealth rates were 23.3% in coverage + payment parity states vs. 20.2% (coverage-only) and 19.1% (none), and cross-state limits were associated with 14.9% vs. 17.8% use (Δ = −2.9 pp) [19]; at the health-system level, parity mandates were linked to a +2.5 pp telemedicine share in Q1-2023 [23]; a broader Medicaid coverage policy bundle showed +6.01 pp live-video use and +11.12% “always able to access needed care” [20]. Null associations appear for ED telemedicine receipts in 2016–2017 [17] and for hospital telehealth capability in 2019–2020 [21]. For commercially insured workers, parity increased telehealth and outpatient visits, though precise magnitudes were NR in OA tables [25].

**Figure 2:**
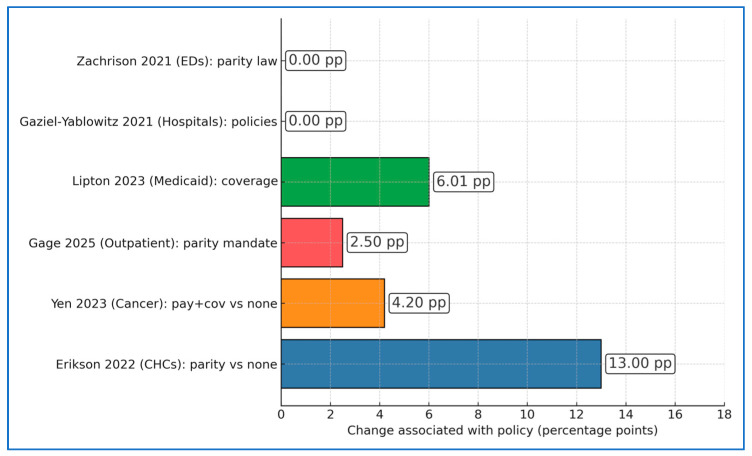
Policy-associated percentage-point changes (utilization/access) across studies. Values represent effect estimates reported by included studies. pp represents percentage points. Asterisks denote statistically supported effects (95% CI excludes 0 or *p* < 0.05 as reported); ‘NS/NR’ indicates not significant or not reported [17,18,19,20,21,23].

Figure 2 compares policy-linked percentage-point (pp) increases in utilization/access across studies: Erikson 2022 [18] shows CHC telehealth use 42% vs. 29% in April 2021 for states with vs. without payment parity (Δ +13.0 pp); Yen 2023 [19] finds adjusted rates 23.3% in coverage + payment parity states vs. 19.1% in no/unspecified parity (Δ +4.2 pp); Gage 2025 [23] estimates a +2.5 pp parity effect for outpatient telemedicine in Q1-2023; Lipton 2023 [20] (Medicaid) reports +6.01 pp higher live-video use under coverage requirements (and +11.12 pp for “always able to access care,” not plotted). In contrast, Gaziel-Yablowitz 2021 [21] (hospital adoption) and Zachrison 2021 [17] (ED telemedicine) observed no associations after adjustment (shown as 0.00 pp). Taken together, policy generosity commonly maps to +2.5–13.0 pp higher use in ambulatory contexts, while some pre-COVID adoption settings exhibit null policy signals.

### 3.3. Quality, Access, and Equity Outcomes

Table 3 aggregates outcomes beyond raw utilization. Quality: payment parity was significantly associated with better hypertension medication adherence (e.g., +0.43–0.46 pp for MPR ≥ 0.8/≥1.0 and +2.14 days’ supply), while coverage parity alone increased days’ supply only [22]. Equity/modality: by March 2023, telemedicine constituted 29% of mental-health and 21% of SUD visits, with an overall +2.5 pp parity effect [23]; oncology cohorts experienced lower use where cross-state limits applied (14.9% vs. 17.8%, Δ = −2.9 pp) [19]. Workforce/access: FQHC interviews described low Medicaid reimbursement (i.e., non-parity) as worsening behavioral-health staffing and access inequities [24]. Access (policy bundle): +11.12% “always able to access needed care” and +6.01 pp shift to live video [20]. Spending: direct cost estimates remain sparse/NR; among privately insured workers, parity increased telehealth and total outpatient visits without clear in-person declines reported in OA summaries [25].

**Figure 3:**
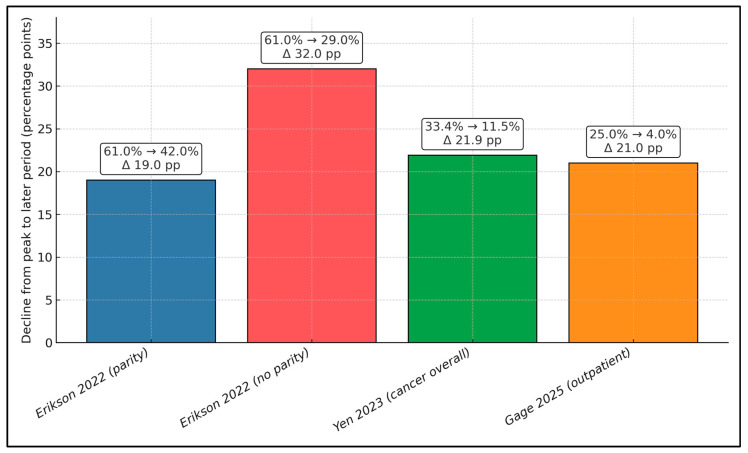
Decline from peak pandemic telehealth share to later periods; Asterisks denote statistically supported changes where uncertainty was reported; otherwise, values are descriptive [18,19,23].

Figure 3 quantifies the decline from peak pandemic telehealth shares to later baselines across settings. In CHCs, usage fell from 61% at peak to 42% in parity states (−19.0 pp) versus 29% without parity (−32.0 pp), suggesting parity buffered the fall-off (Erikson 2022 [18]). In oncology, Yen 2023 [19] shows 33.4% → 11.5% (−21.9 pp) within one year of diagnosis during 2020–2021. For all outpatient encounters, Gage 2025 [23] reports 25.0% → 4.0% (−21.0 pp) by March 2023. Despite broad retrenchment from early peaks, late-period telehealth levels remained substantially higher where supportive reimbursement was present (e.g., 42% vs. 29% in CHCs), indicating a stabilizing influence of parity on sustained telehealth availability.

### 3.4. Spending and Sustainability

Table 4 provides numeric details by study: CHC telehealth peaked at 61% (April 2020) and stabilized at 42% (parity) vs. 29% (non-parity) by April 2021 (Δ = 13.0 pp), with OR 1.74 (95% CI 1.49–2.03) favoring parity [18]; oncology patients peaked at 33.4% telehealth, declining to 11.5% by March 2021, with adjusted rates 23.3% (coverage + payment), 20.2% (coverage-only), 19.1% (none), and cross-state restrictions reducing use by −2.9 pp (14.9% vs. 17.8%) [19]; outpatient EHR data linked parity to +2.5 pp telemedicine in Q1-2023 and late-period shares of 29% (mental health) and 21% (SUD) [23]; ED analyses showed 53% of EDs using telemedicine in 2016 (state range 13–89%) with no parity association [17], and 73% of hospitals had ≥1 telehealth capability with no state-policy effects after adjustment [21]; Medicaid coverage requirements associated with +6.01 pp live-video use and +11.12% access [20]; among Medicare beneficiaries with cardiovascular conditions, payment parity improved adherence (MPR thresholds +0.43–0.46 pp; +2.14 days’ supply), while coverage parity increased days supplied only [22]; qualitative FQHC findings highlighted non-parity as destabilizing (numeric NR) [24]; in privately insured workers (*n* = 29,204; 1,051,344 person-months), parity increased telehealth and total outpatient visits, with magnitudes NR in OA abstracts [25].

## 4. Discussion

### 4.1. Summary of Evidence

The systematic review indicates that sustaining more generous reimbursement for virtual encounters is plausibly one mechanism by which states have preserved higher post-PHE telehealth levels in ambulatory settings. Evidence outside oncology and community health centers also points to concrete access/process gains that parity could help maintain. For example, large observational cohorts show that telemedicine visits are completed more often than in-person encounters—an operational advantage that aligns with the “visit completion” pathway posited for sustained use and timely follow-up [26]. Parallel population-based analyses demonstrate fewer missed appointments with telemedicine overall, while also flagging that some disparities in appointment adherence persist, underscoring the need for payment design to be paired with equity safeguards [27]. These results are directionally consistent with the review’s finding that payment parity buffered the fall-off in use after initial pandemic peaks, particularly for ambulatory care contexts in which logistics and time costs are salient.

Modality provisions embedded in state law likely mediate parity’s effect size. Empirical work from Washington State leveraging new audio-only claim modifiers shows that telephone-only services were used predominantly for behavioral health and perinatal care and were more common among older and Medicare-insured patients—groups for whom device, bandwidth, and usability barriers to video are well documented [28]. National survey data from 2022 similarly show that the audio-only share remains meaningful among adults with lower digital access, while convenience and time-saving reasons for choosing telemedicine are widespread, implying that parity statutes explicitly including audio-only reimbursement may deliver larger access gains for digitally marginalized populations [29]. Together, these studies support the review’s observation that payment parity statutes that specify modality scope—especially inclusion of audio-only—map to higher sustained utilization in settings where video feasibility is uneven.

Quality-of-care implications of expanded, reimbursed telemedicine appear nuanced but generally reassuring for primary care. A recent pediatric primary care cohort found that telephone/video visits were associated with less prescribing and fewer imaging/lab orders than in-person visits, with only modest increases in downstream in-person and emergency department follow-up, and no increase in hospitalizations [30]. These results align with the mechanism of adequately reimbursed virtual touchpoints substituting for some low-intensity encounters without clear evidence of widespread overuse or safety concerns—provided there are guardrails for clinical appropriateness. When interpreted alongside parity-linked adherence improvements in chronic disease management within this review, the external literature suggests that payment signals encouraging virtual follow-up can support guideline-concordant monitoring without degrading outcomes.

At the same time, classic work on direct-to-consumer telehealth reminds policymakers that reimbursement parity can, in some contexts, increase total utilization rather than purely substitute for in-person care. Pre-pandemic claims analyses of employer-sponsored plans found that new access induced by telehealth contributed to higher overall spending for certain acute conditions, primarily via additional low-acuity encounters [31]. Those findings, while predating contemporary hybrid models, reinforce the importance of coupling parity with value-oriented benefit design, episode-level appropriateness criteria, and monitoring of additive use—especially outside behavioral health and chronic care, where substitution appears stronger in recent parity-era studies summarized in this review.

Licensure and cross-state practice policies intersect with reimbursement in shaping realized access under parity. During the PHE, licensure waivers substantially increased out-of-state tele-mental-health connections, particularly facilitating continuity for established patients; effects varied by urbanicity and were often larger where interstate compacts eased cross-border frictions [32]. Among Medicare beneficiaries, interstate telehealth use surged in 2020 under licensure flexibilities, with many visits occurring across neighboring states—patterns that later softened as waivers expired [33]. These studies complement the review’s finding that parity advantages are attenuated by cross-state restrictions; payment signals alone are insufficient when licensure barriers limit provider supply or continuity across borders.

Finally, distributional effects merit emphasis. National Medicare analyses show that, after adjustment for geography and other covariates, Black and Hispanic beneficiaries had fewer telemedicine visits per capita than White beneficiaries during the second pandemic year—despite higher unadjusted use in high-telemedicine regions [34]. Combined with survey evidence on modality divides and the Washington State claims audit of audio-only coding, these results clarify why parity features such as explicit audio-only coverage, mental-health carve-ins, and site-of-service flexibility are likely necessary but not sufficient to close gaps. In practice, parity may stabilize the supply of telehealth, but equitable uptake will depend on complementary digital inclusion, language access, and benefit designs that minimize cost-sharing for low-income populations—design elements that several studies in this review identified as moderators of parity-associated gains.

In clinical practice, stable, predictable reimbursement at parity supports sustained hybrid models—protecting telehealth capacity after the pandemic surge. Clinicians in behavioral health and chronic disease management can anticipate small-to-moderate utilization gains (≈+2.5 to +13.0 pp) when parity is in force, with signals of improved medication adherence in cardiovascular care. Including audio-only within parity frameworks can extend access to patients facing bandwidth or device barriers, while organizations serving rural and low-income populations may leverage parity to justify staffing, training, and workflow investments that reduce no-shows and improve visit completion.

From a sustainability standpoint, payment parity should be judged not just through visit counts but also based on its impact on access and clinical outcomes for chronic conditions. In the open-access literature synthesized here, the clearest outcome signal is improved medication adherence among adults with hypertension under payment parity [22], alongside self-reported gains in the ability to access needed care under broader coverage reforms [20]. We did not identify parity-specific outcome studies for other high-priority conditions such as diabetes, depression, or serious mental illness, despite their prominence in telehealth policy debates, which highlights important gaps for future research.

Taken together, these findings suggest several practical priorities for evaluators and policymakers considering the sustainability of telehealth under payment parity. First, audio-only coverage decisions should be explicitly monitored, because modality rules appear to shape who can realistically benefit from telehealth. Second, parity provisions need to be coordinated with licensure reciprocity and interstate compacts so that reimbursement incentives are not undermined by provider-supply constraints or cross-state frictions. Third, given the limited cost evidence, future evaluations should prospectively align parity reforms with pre-specified spending outcomes (for example, per-member-per-month spending, episode-level cost, and out-of-pocket payments) to clarify whether observed utilization increases represent substitution, additive use, or targeted access improvements.

### 4.2. Limitations

The evidence was observational and heterogeneous, so residual confounding from concurrent policies (such as coverage mandates or licensure compacts) is likely. Policy operationalization varied across studies, and misclassification of statute timing or scope is possible. Outcomes and denominators differed (claims vs. EHR vs. surveys), which precluded meta-analysis. We restricted inclusion to open-access full texts to maximize transparency and replicability. This may under-represent policy and payer studies in subscription journals and introduces potential selection bias. To mitigate this, we attempted to obtain paywalled full texts by searching for author-posted versions and by contacting corresponding authors; nonetheless, unavailable full texts remained a reason for exclusion. As with all evidence syntheses, unpublished or inaccessible null results may remain under-captured. Because we did not have full-text access to paywalled papers, we were unable to perform a formal sensitivity analysis that counted how many such studies would otherwise have met our eligibility criteria; instead, we transparently mark this as a potential source of selection bias. Finally, generalizability to self-funded ERISA plans and to statutory changes enacted after 2025 is uncertain.

## 5. Conclusions

Payment parity appears to help “lock in” telehealth for ambulatory care, most notably in behavioral health and chronic disease follow-up, with ancillary gains in adherence and perceived access. However, parity alone is not a panacea: cross-state practice frictions, digital inclusion gaps, and unclear spending effects temper expectations. Policymakers should pair parity with licensure reciprocity and quality safeguards, and researchers should prioritize robust, parity-specific cost and equity evaluations. In evaluating sustainability, future work should treat not only telehealth volume but also changes in access, clinical outcomes, and patient-reported experience as core endpoints.

## Figures and Tables

**Table 1:** Study and policy characteristics.

#	Study (Year)	Setting/Population	Policy Exposure	Design and Data Source	Period	N
1	Zachrison KS et al., 2021 [17]	U.S. Emergency Departments	Presence/duration of payment parity law	Cross-sectional and panel regression; national ED survey	2016–2017	4418
2	Erikson C et al., 2022 [18]	Community Health Centers; privately insured	Payment parity vs. none (state law)	Logistic regression; FAIR Health data	2019–2021	6598
3	Yen TWF et al., 2023 [19]	Oncology patients (5 cancers)	Payment + coverage parity vs. coverage-only vs. none; cross-state policy limits	ITS and multivariable models; EHR	March 2019–March 2021	110,461 total; 53,982
4	Lipton BJ et al., 2023 [20]	National; claims	Coverage parity and related requirements; reports parity-associated outcomes	Quasi-experimental	2011–2020	*n* = 4492 Medicaid; *n* = 15,581 private
5	Gaziel-Yablowitz M et al., 2021 [21]	U.S. hospitals (policy landscape)	State reimbursement/coverage parity prevalence	Cross-sectional; AHA IT survey	2019–2020	2923 hospitals
6	Zhang D et al., 2024 [22]	National; adults with hypertension	Payment parity vs. coverage parity vs. none	DiD; claims	2016–2022	353,220
7	Gage AD et al., 2025 [23]	National health systems (Healthjump EHR)	Payment parity mandates	DiD; system-month panel	January 2019–March 2023	498
8	Porteny T et al., 2025 [24]	NYC FQHC staff/leadership	Medicaid reimbursement levels; implications of (non-)parity	Qualitative interviews	2022–2024	56 interviews, 6 FQHCs
9	Zhang Z et al., 2025 [25]	Commercially insured workers	Payment parity status	DiD; claims	2019–2024	29,204 workers; 1,051,344 person-months

ED—emergency department; FQHC—Federally Qualified Health Center; EHR—electronic health record; ITS—interrupted time series; DiD—difference-in-differences; N—sample size/observations.

**Table 2:** Utilization outcomes.

Study	Outcome and Measure	Data and Findings
Zachrison et al., 2021 [17]	ED telemedicine adoption/use	No association between state payment parity presence/duration and ED telemedicine receipt (2016–2017).
Erikson et al., 2022 [18]	CHC telehealth use (site-month; OR)	Telehealth OR 1.740 (*p* < 0.001) for CHC sites in payment-parity states in 2021; usage peak 61% (April 2020); 29% vs. 42% of sites using telehealth in April 2021 without vs. with parity.
Yen et al., 2023 [19]	Oncology telehealth use (adjusted rates)	Privately insured in payment + coverage parity states used telehealth 23.3% vs. 20.2% (coverage-only) and 19.1% (no/unspecified); cross-state limits 14.9% vs. 17.8% without limits.
Lipton et al., 2023 [20]	Telemedicine use/access	Medicaid coverage requirements associated with increase in live video use +6.01% and increased access to care +11.12% (policy bundle including parity/coverage).
Gage et al., 2025 [23]	System-level telemedicine share	Payment parity mandates associated with +2.5 percentage-point telemedicine use in Q1-2023 (DiD).
Zhang Z et al., 2025 [25]	Outpatient service utilization (commercial plans)	Direction/significance reported for telehealth and outpatient visits under parity.

OR—odds ratio; CHC—community health center; ED—emergency department. Interpretation note: Descriptive percentages are reported as stated in the source study. Inferential language is used only when the included study reported a statistical comparison (effect estimate with CI and/or *p*-value).

**Table 3:** Quality, equity, workforce, and spending outcomes.

Study	Domain	Key Findings
Zhang D et al., 2024 [22]	Quality (HTN medication adherence)	Payment parity significantly associated with improved adherence; coverage parity alone not significant. Effect sizes reported as significant
Gage et al., 2025 [23]	Equity and case-mix	By March 2023, 29% of mental-health visits and 21% of SUD care delivered via telemedicine; parity mandates associated with higher use overall (DiD).
Yen et al., 2023 [19]	Equity modifier (policy interactions)	Telehealth rates were 15%+ lower where cross-state policies had limits (14.9% vs. 17.8%), indicating access friction despite parity.
Porteny et al., 2025 [24]	Workforce and equity (FQHC qualitative)	Staff perceived low Medicaid telehealth reimbursement as exacerbating mental-health workforce shortages and access inequities; parity seen as stabilizing.
Lipton et al., 2023 [20]	Access	Policy bundle including coverage parity associated with +11.12% access to care; modality shifts toward live video +6.01%.
Zhang Z et al., 2025 [25]	Spending	Direct spending outcomes NR in OA tables; utilization shifts suggest possible substitution effects; requires further OA cost studies.

SUD—substance use disorder; FQHC—Federally Qualified Health Center; DiD—difference-in-differences; OA—open access. Interpretation note: Descriptive percentages are reported as stated in the source study. Inferential language is used only when the included study reported a statistical comparison (effect estimate with CI and/or *p*-value).

**Table 4:** Study findings.

Study	Setting and Population (N)	Policy Exposure(s)	Outcome Metric(s)	Quantitative Findings
Erikson et al., 2022 [18]	U.S. Community Health Centers (site-level; N = NR)	State payment parity for private insurers vs. no parity	% of all CHC visits via telehealth; policy associations	Peak telehealth share 61% in April 2020; by April 2021 42% in parity states vs. 29% in non-parity (Δ = 13.0 pp). Adjusted OR for telehealth in parity vs. non-parity: 1.74 (95% CI 1.49–2.03).
Yen et al., 2023 [19]	Newly diagnosed cancer, U.S. (N = 110,461 overall; parity analyses N = 53,982 since March 2020)	Coverage + payment parity vs. coverage-only vs. no/unspecified parity; Cross-state restrictions vs. none	Adjusted telehealth use rate (%)	Peak 33.4% (April 2020); 11.5% by March 2021. Adjusted rates: 23.3% (coverage + payment), 20.2% (coverage-only), 19.1% (no/unspecified); parity advantage +4.2 pp vs. none. Cross-state restrictions associated with lower use, 14.9% vs. 17.8% (−2.9 pp).
Gage et al., 2025 [23]	U.S. outpatient EHR data (Healthjump); all ages (N = NR)	Presence of payment parity mandate by state	% outpatient telemedicine visits; subgroup levels	Telemedicine share rose to 25% (April 2020) and stabilized at 4% (March 2023). Payment parity associated with +2.5 pp telemedicine use in Q1-2023. By specialty in March 2023: mental health 29%, SUD 21%. Urban areas had ~2.4× higher telemedicine share than rural.
Zachrison et al., 2021 [17]	U.S. EDs (N = 4418 EDs; 82% response)	Any telemedicine parity law vs. none (state level)	ED receipt of telemedicine (yes/no)	53% of EDs received telemedicine in 2016; state range 13–89%. Parity laws not associated with ED telemedicine use or adoption.
Gaziel-Yablowitz et al., 2021 [21]	U.S. hospitals (N = 2923 acute care hospitals)	Commercial and Medicaid reimbursement policies (multiple types)	Hospital telehealth capability (≥1 service)	73% had ≥1 telehealth capability. In multivariable analysis, no statewide policy was associated with adoption (organizational factors dominated).
Lipton et al., 2023 [20]	U.S. adults < 65: Medicaid (*n* = 4492) and privately insured (*n* = 15,581)	State Medicaid telehealth coverage requirements; private coverage policies	Live video use; “always able to access needed care”	Medicaid coverage requirements +6.01 pp live video use; +11.12 pp “always able to access needed care.” Private-plan coverage not significantly associated.
Zhang et al., 2024 [22]	U.S. Medicare beneficiaries with cardiovascular conditions (N = 353,220)	Payment parity and coverage parity (state)	Medication adherence: MPR ≥ 0.8/≥1.0; days’ supply	Payment parity associated with +0.43 pp (MPR 0.8–1.2) and +0.46 pp (MPR ≥ 1.0) adherent; +2.14 days’ supply. Coverage parity: +2.13 days supply only.
Porteny et al., 2025 [24]	New York City FQHC staff (qualitative; 56 interviews)	Medicaid reimbursement levels (perceptions of non-parity)	Themes: workforce, access, sustainability	Staff perceived low Medicaid telehealth reimbursement as worsening workforce shortages and access inequities (esp. behavioral health); numeric effect NR.
Zhang et al., 2025 [25]	Commercially insured workers (N = 29,204; 1,051,344 person-months)	Payment parity (state)	Telehealth and total outpatient visits	Parity increased telehealth and total outpatient visits without notable rise in in-person visits; precise magnitudes NR in abstract (full OA indicates positive effects).

N—sample size/observations; pp—percentage points; OR—odds ratio; CI—confidence interval; EHR—electronic health record; ED—emergency department; FQHC—Federally Qualified Health Center; OA—open access; NR—not reported; MPR—Medication Possession Ratio; SUD—substance use disorder.

## Data Availability

No new data were created or analyzed in this study.
